# Distinct Gut and Skin Microbiomes of a Carnivorous Caecilian Larva (*Ichthyophis bannanicus*) Show Ecological and Phylogenetic Divergence from Anuran Tadpoles

**DOI:** 10.3390/microorganisms13102405

**Published:** 2025-10-21

**Authors:** Amrapali Prithvisingh Rajput, Dan Sun, Shipeng Zhou, Madhava Meegaskumbura

**Affiliations:** 1 Guangxi Key Laboratory of Forest Ecology and Conservation, College of Forestry, Guangxi University, 100 Daxue Road, Nanning 530004, China; amrapali20@gmail.com; 2 Pinglu Canal and Beibu Gulf Coastal Ecosystem Observation and Research Station of Guangxi, Guangxi Key Laboratory of Marine Environmental Disaster Processes and Ecological Protection Technology, College of Marine Sciences, Beibu Gulf University, Qinzhou 535000, China; 3 Key Laboratory of Bio-Resources and Eco-Environment of Ministry of Education, College of Life Sciences, Sichuan University, Chengdu 610065, China; zsp_andy@163.com

**Keywords:** gut–skin axis, microbiome, *Ichthyophis bannanicus*, Gymnophiona, larval microbial ecology, 16S rRNA gene

## Abstract

The amphibian microbiome plays a vital role in host health, yet the bacterial communities of caecilians (Order: Gymnophiona) remain largely uncharacterised. We investigated this by providing the first characterisation of the gut and skin microbiome of larval *Ichthyophis bannanicus*, a carnivorous caecilian, using 16S rRNA gene metabarcoding. Our analyses show distinct communities between the faecal samples and skin, with significant enrichment of *Laribacter* in faeces and *Flavobacterium* on skin. Despite significant variation in their community structures, the core genera *Escherichia-Shigella* were shared between both regions, suggesting similar microbial exchange in the aquatic environments. Skin bacterial diversity exhibited relatively higher richness, but lower evenness than that of faeces. Further, the skin bacterial community exhibited more complex interactions, suggesting stronger resilience to changes. The relationships and interactions of skin and faecal bacterial communities suggest their interactive effects on the host’s overall health. Compared with anuran tadpoles, the *I. bannanicus* larval microbiome showed taxonomic overlap, but possessed certain unique core bacteria. This work on an understudied amphibian lineage is foundational, highlighting how diet, phylogeny, and aquatic environment shape microbial communities and informing future research into amphibian health and disease.

## 1. Introduction

The bacterial microbiome plays an important role in the health of its host [1]. Its composition is influenced by the host’s evolutionary background and life history [2,3]. Amphibians, with their diverse ecologies and developmental pathways, provide an opportunity to study these interactions [4,5]. The microbial communities found in their gut and skin are known to impact immunity against pathogens [6]. However, microbiome research has primarily focused on frogs and salamanders, while the order Gymnophiona (caecilians, 3% of amphibian species) remains poorly understood [7,8,9].

Caecilians are elusive, often fossorial or aquatic animals [10]. Their secretive nature means that basic aspects of their biology are still being described. This is especially true for the Kohtao striped caecilian, *Ichthyophis bannanicus*. This species inhabits parts of Southeast Asia and has a prolonged, two-year aquatic larval phase before it metamorphoses into a terrestrial adult [11,12]. The diet of these larvae is carnivorous. This contrasts with the mainly herbivorous or omnivorous feeding habits of most anuran tadpoles, offering a point of comparison.

It is known that an organism’s diet, environment, and developmental stage influence its microbiome [13,14,15,16]. Amphibian skin is a rich habitat for bacteria that can play protective roles [17,18]. The gut microbiome changes to meet new nutritional demands, especially during metamorphosis [19]. For aquatic larvae like *I. bannanicus*, the gut and skin are constantly exposed to the same environment. Defecation into the water and skin shedding could allow for microbial exchange between these two body sites. This interaction between gut and skin bacteria may be important in maintaining the host’s health [20,21]. However, previous studies mainly focused on the microbial diversity and composition in the gut and skin of adult amphibians [22,23].

Here, we use 16S rRNA gene sequencing to investigate the gut and skin microbiota of larval *I. bannanicus*. We also compare these communities with those of anuran tadpoles. We focus on three questions. First, how do the bacterial communities of the skin and gut overlap? Second, what structural patterns exist within the skin and gut microbiomes? And third, how does the microbiota of a carnivorous caecilian larva compare with that of herbivorous anuran tadpoles?

## 2. Materials and Methods

### 2.1. Sample Collection

The larvae of *I. bannanicus* (N = 15) were collected from the wild but maintained in captivity and fed with chirnomid larvae. To reduce stress, animals were maintained in a glass holding tank in aged water, which was changed regularly. The holding tank had several stones as a climbing surface for them to rest outside the water or climb onto as they developed. The microbial samples from each individual were collected (under sterile conditions) within seven days of being transferred to the lab. They were fed to induce defecation. Tadpoles of Anurans (*Hoplobatrachus chinensis*: N = 7; *Microhyla heymonsi*: N = 2; *Hyla sanchiangensis*: N = 3; *Duttaphrynus melanostictus*: N = 5; *Polypedatus megacephalus*: N = 2) were collected from wild habitats between June and August in 2019. After collecting skin swab samples, the larvae were placed in sterile water. As soon as the animals defecated, the sample was collected using a sterile dropper. Vials containing samples were immediately frozen at −80 °C until DNA extraction.

### 2.2. DNA Extraction, PCR Amplification and Sequencing

Following the manufacturer’s instructions, we extracted bacterial genomic DNA from the faecal and skin samples using PowerSoil DNA Isolation Kits and DNeasy Blood & Tissue Kits (QIAGEN, Hilden, Germany). We assessed the quality and quantity of the extracted DNA using agarose gel electrophoresis (1.0%), a Qubit fluorometer (Thermo Fisher Scientific, Wilmington, DE, USA), and a NanoDrop spectrophotometer (Thermo Fisher Scientific, Wilmington, DE, USA).

The V3–V4 hypervariable regions of the 16S rRNA gene were amplified using the universal bacterial primers 341F (CCTAYGGGRBGCASCAG) and 806R (GGACTACNNGGGTATCTAAT). Each polymerase chain reaction contained 15 µL of Phusion High-Fidelity PCR Master Mix (New England Biolabs, Ipswich, MA, USA), 0.2 µM of each primer, and 10 ng of template DNA. The thermal cycling protocol began with an initial denaturation at 98 °C for 1 min using T100 PCR (BIO-RAD, Hercules, CA, USA). This was followed by 30 cycles of denaturation at 98 °C for 10 s, annealing at 50 °C for 30 s, and extension at 72 °C for 30 s. A final extension step was performed at 72 °C for 5 min. We verified the PCR products on a 2% agarose gel.

The sequencing libraries were generated using TruSeq^®^ DNA PCR-Free Sample Preparation Kit (Illumina, San Diego, CA, USA) following the manufacturer’s protocol, followed by the addition of index codes. Library quality was evaluated on a Qubit@ 2.0 Flurometer (Thermo Fisher Scientific, Waltham, MA, USA) and Agilent Bioanalyzer 2100 system (Agilent Technologies, Santa Clara, CA, USA). The library was sequenced on an Illumina NovaSeq platform (Illumina, San Diego, CA, USA), generating 250 bp paired-end reads. Paired-end reads were assigned to the samples based on their unique barcode, truncated by cutting off the barcode and primer sequence. The paired-end reads were further merged using FLASH v 1.2.11, and then the splicing sequences were filtered using fastp v0.23.1 to obtain high-quality data [24]. The filtered data were compared with the SILVA database (https://www.arb-silva.de/, accessed on 5 June 2023) using the UCHIME algorithm to detect chimaera sequences [25], then denoised using DADA2 in the QIIME2 v 2020.06, to obtain initial Amplicon Sequence Variants (ASVs).

The SILVA database was used to annotate the taxonomic information. The final 12 574 ASVs were retained after removing the sequences classified to archaea, mitochondria, chloroplasts, and unassigned ASVs. The ASV abundance was normalised using the standard sequence number corresponding to the sample with the fewest sequences. The multiple sequence alignment was performed using QIIME2, to study the phylogenetic relationship of each ASV.

### 2.3. Data Analysis

The shared and unique classification and ASVs between the skin and gut of *I. bannanicus* were visualised in Venn diagrams that were generated using the “ggvenn” package in R v 4.2.2 [26]. To compare the bacterial community composition between the skin and faeces of *I. bannanicus*, we calculated Bray–Curtis and UniFrac distances at the ASV level, then performed an analysis of similarity (ANOSIM), and principal coordinate analysis (PCoA) [27]. Linear discriminant analysis (LDA) and effect size (LEfSe) identified differential bacterial taxa, considering statistical significance and biological relevance [28]. The core ASVs were defined as ASVs that were present in all samples from caecilians, and represented ≥0.1% of relative abundance [29]. We performed PICRUSt analysis to predict the functional attributes of microbiomes on faeces and skin of *I. bannanicus*.

Network analysis was conducted to show the co-occurrence and co-exclusion relationships among bacterial communities on faeces and skin of *I. bannanicus* [30]. We selected ASVs that were present in all samples and had >0.1% of relative abundance. The networks of bacterial interactions were constructed based on Spearman correlation coefficients (ρ) > 0.7 and adjusted *p*-values < 0.05, and visualised in Gephi v 0.9.7 [31]. Various network topological properties (the number of nodes and edges, modularity, clustering coefficient, centrality, average path length, network diameter, average degree, and graph density) were calculated. The proportion of realised links from all possible connections in the network was correlated with network complexity [32].

To understand the associations of bacterial community between skin and faeces of *I. bannanicus*, we firstly calculated the bacterial richness and evenness using the package “vegan”, and compared these metrics between the two regions. Secondly, we used linear regression to analyse the relationships between their bacterial diversity and community composition similarity. Finally, we constructed the co-occurrence of skin and faecal bacterial communities in Gephi v 0.9.7. We analysed the shared and unique classification and ASVs between *I. bannanicus* and anuran species to understand the ecological divergence between the two orders.

## 3. Results

### 3.1. Distinct Gut and Skin Bacterial Communities in I. bannanicus

**Figure 1:**
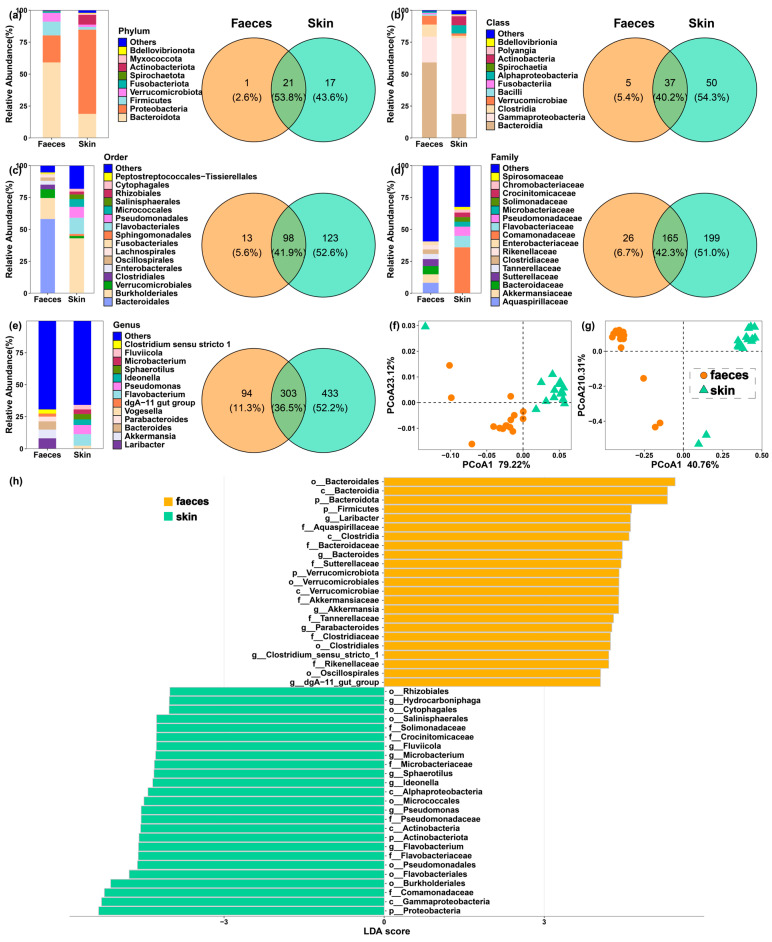
Taxonomic composition and differences between faecal and skin microbiomes of *I. bannanicus* larvae. Relative abundance of bacterial community and shared taxa between faeces and skin: (**a**) at phylum level; (**b**) at class level; (**c**) at order level; (**d**) at family level; (**e**) at genus level. Principal coordinate analysis (PCoA) based on: (**f**) unweighted UniFrac distances; (**g**) Bray–Curtis distances. (**h**) Discriminant bar chart of the different effects of important taxa on bacterial community composition. The number of differences in taxonomic compositions between faeces and skin was greater at finer levels.

The faecal and skin samples from larval *I. bannanicus* were both dominated by the phyla Bacteroidota and Proteobacteria (Figure 1a,b). At a finer taxonomic resolution, however, the communities were quite different (Figure 1c–e). Bacteroidales was the most abundant order in faecal samples (58.1% mean relative abundance), whereas Burkholderiales was most abundant on the skin (43.1%). These differences continued down to the genus level. For example, the genera *Laribacter* and *Akkermansia* were common in faecal samples but were rare on the skin.

Beta diversity analyses confirmed a strong and significant separation between the faecal and skin bacterial communities (ANOSIM on unweighted UniFrac: *R* = 0.69, *p* = 0.001; Bray–Curtis: *R* = 0.99, *p* = 0.001). The principal coordinate analysis plots show this clear separation between the two sample types (Figure 1f,g).

**Figure 2:**
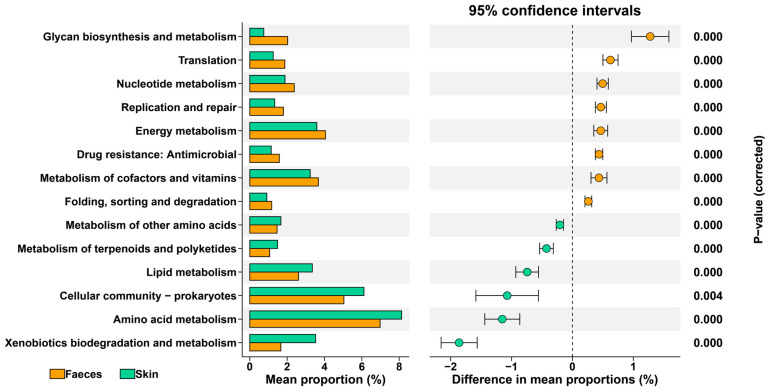
Functional differences in microbial communities on the faecal and skin of *I. bannanicus* larvae.

We used LEfSe to identify the specific bacterial taxa driving these differences (Figure 1h). The analysis showed that the phyla Bacteroidota, Firmicutes, and Verrucomicrobiota were significantly enriched in faecal samples. In contrast, Proteobacteria and Actinobacteriota were enriched in skin samples. *Laribacter*, *Bacteroides*, and *Akkermansia* were among the taxa significantly more abundant in faeces at the genus level. The skin was enriched considerably with genera such as *Flavobacterium*, *Pseudomonas*, and *Ideonella*. The identified distinct bacteria could be reflected on their functional attributions (Figure 2). The relatively richer in cellular community prokaryotes and amino acid metabolism might be involved in the genus *Pseudomonas*.

### 3.2. Core Microbiome and Co-Occurrence Networks

The core microbiome of the faeces and skin was also distinct (Table 1). The main core members in the faeces included an ASV assigned to *Laribacter* (ASV9), another to *Akkermansia* (ASV12), and several unclassified Bacteroidota. The skin’s core microbiome was dominated by an ASV from the genus *Flavobacterium* (ASV6) and the family Comamonadaceae affiliated bacteria (ASV16 and ASV17). Only one core ASV, assigned to *Escherichia–Shigella* (ASV115), was found in the faecal and skin samples.

**Figure 3:**
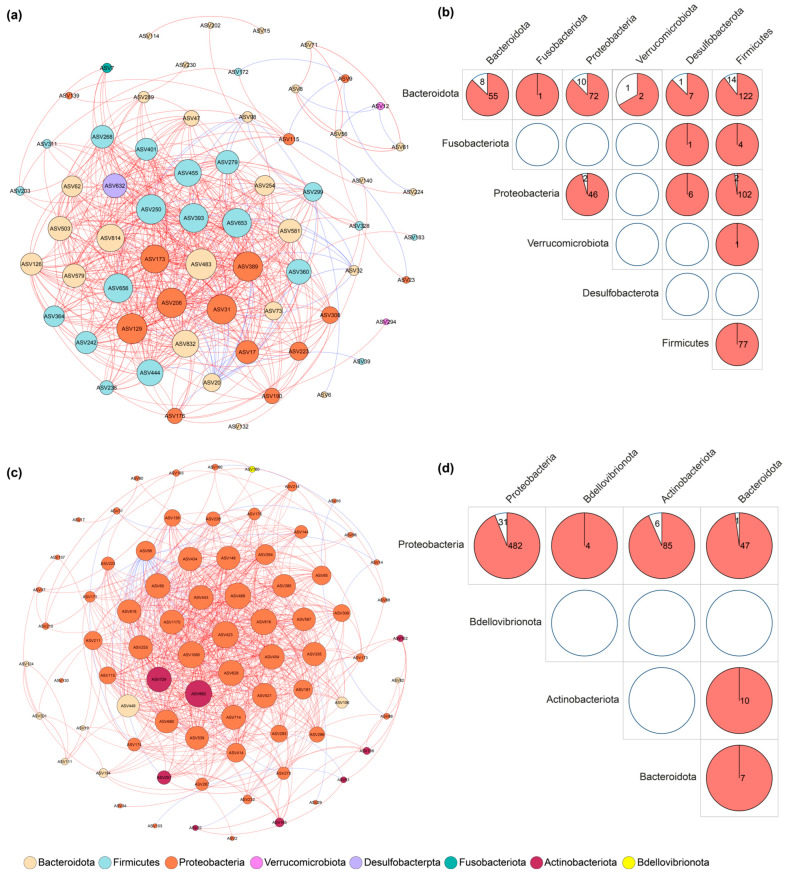
Co-occurrence networks of faecal and skin bacterial communities. (**a**) Bacterial co-occurrence network based on faecal samples. (**b**) The bacterial compositions and their correlations in the faecal bacterial network. (**c**) Bacterial co-occurrence network based on skin samples. (**d**) The bacterial compositions and their correlations in the skin bacterial network. Nodes represent ASVs; edges represent strong positive (red) or negative (blue) Spearman correlations (ρ > 0.7, *p* < 0.05). Dominant phyla are colour coded. Numbers in circles represent the positive and negative relationships between members from different bacterial phyla.

The co-occurrence network constructed from the skin bacteria was larger and more complex than the network from the faecal bacteria, containing more nodes and edges and a higher modularity score (Figure 3, Table 2). Within the faecal network, interactions were most numerous among bacterial ASVs from the phyla Firmicutes, Proteobacteria, and Bacteroidota (Figure 3b). In the skin network, Proteobacteria was the most connected phylum (Figure 3d). Interestingly, several of the most abundant core ASVs, including *Flavobacterium* (ASV6) on the skin and *Laribacter* (ASV9) in the faeces, were not identified as highly connected nodes in their respective networks.

### 3.3. The Diversity of Skin and Faecal Microbiome and Their Relationships

**Figure 4:**
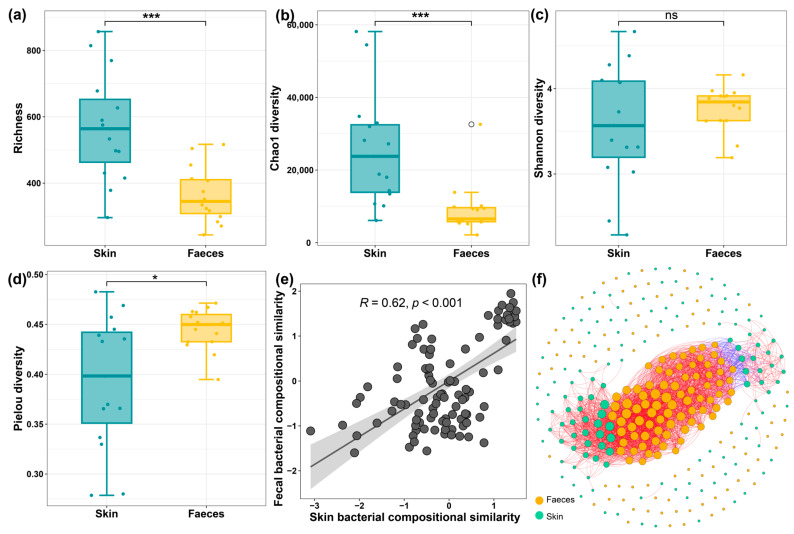
Alpha diversity of skin and faecal bacterial communities (**a**–**d**); (**e**) Linear regression of Beta diversity of faecal and skin bacterial community similarity based on the Bray–Curtis distance; (**f**) Co-occurrence network of skin and faecal bacterial communities The bacterial composition is strongly correlated with the variations in the skin bacteriome. The ns, * and *** represent the *p* > 0.05, *p* < 0.05 and *p* < 0.001. The red lines represent positive correlations; the blue lines represent the negative correlations.

The alpha diversity metrics showed that bacterial richness and Chao1 diversity were significantly higher on the skin than in the faeces (Figure 4a,b). Conversely, the faecal bacterial community showed significantly higher evenness (Figure 4c,d). Although we did not find significant relationships of alpha diversity between faeces and skin, the bacterial community composition showed one positive relationship between the two regions, suggesting the bacterial composition in faeces might affect skin microbiota (Figure 4e). Moreover, we observed that numerous skin microbiota were correlated with those in the faecal samples (Figure 4f).

### 3.4. Comparison with Anuran Larval Microbiomes

**Figure 5:**
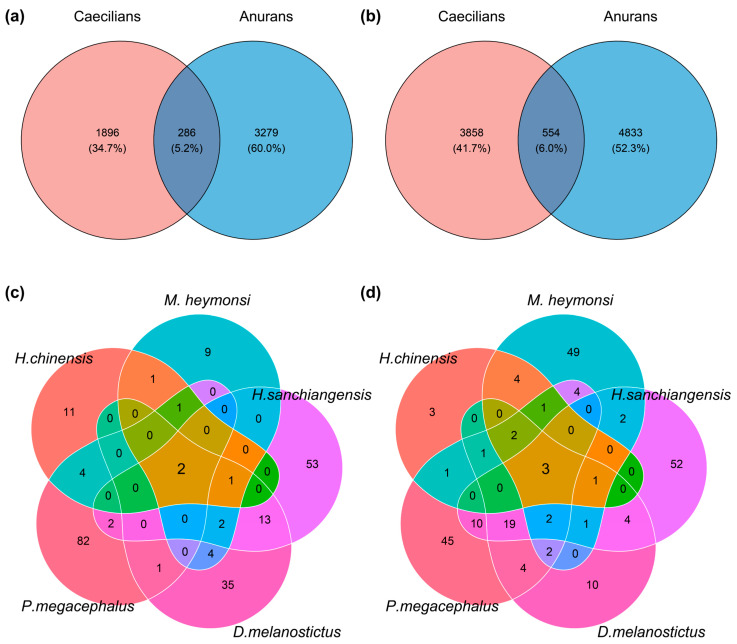
Venn diagrams of shared bacteria between amphibians. Between *I. bannanicus* and anurans: (**a**) faecal ASVs; (**b**) skin ASVs. Across five anuran species: (**c**) faecal ASVs; (**d**) skin ASVs.

When we compared the caecilian data to the anuran tadpoles, we found a degree of overlap (Figure 5). There were 286 ASVs shared in the faecal samples between caecilians and anurans, and 554 shared ASVs on the skin (Figure 5a,b). However, some core bacteria, such as *Laribacter* (ASV9) from faeces and *Flavobacterium* (ASV6) from skin, were not shared (Table 1). Notably, the few ASVs from the genera *Cetobacterium* and *Aeromonas* were shared among the five anuran species (Figure 5c,d), but these shared ASVs were not core members of the *I. bannanicus* microbiome.

## 4. Discussion

Our results suggest that the gut and skin microbiomes of larval *I*. *bannanicus* are distinct communities, each with a unique core membership and structure. This finding is consistent with studies in other amphibians, where different body sites host specialised bacterial assemblages shaped by their local environments [7,15,33]. The skin is in constant contact with the surrounding water, likely leading to a more diverse community influenced by transient environmental microbes [34]. In contrast, the gut provides a more controlled, anaerobic environment geared towards nutrient processing, which would favour a different set of taxa [35].

The dominant bacterial phyla of larval *I. bannanicus*, including Proteobacteria, Bacteroidota and Actinobacteridota on skin, and Bacteroidota, Proteobacteria and Firmicutes on faeces, similar to the compositions on other caecelian species such as *Herpele squalostoma* [33]. Further, the community structure changes were caused by the most anaerobic genus *Laribacter*, *Bacteroides*, *Akkermansia*, *Parabacteroides*, *Clostridium sensu stricto 1*, and *dgA-11 gut group* in the faeces, and the enriched genus *Flavobacterium*, *Pseudomonas*, *Ideonella*, *Sphaerotilus*, *Microbacterium*, *Fluviicola*, and *Hydrocarboniphaga* in the skin.

Despite the clear separation between the gut and skin, some bacteria such as the core membership *Escherichia–Shigella* were shared in the two regions. The result could be attributed to the effects of the environment. Further, the common genus *Escherichia–Shigella* in the gut suggests that the microbial exchange might be affected between the two regions via the water environment. The bacteria from the gut, expelled through defecation, may be able to colonise the skin from the surrounding water. This supports the concept of a “gut-skin axis” in these aquatic larvae, where the two microbial compartments are not entirely isolated [20].

The co-occurrence networks revealed differences between the microbial communities across the skin–gut axis. The skin network had a larger modularity, which is expected, given its higher alpha diversity and exposure to a variable external environment. Higher modularity in ecological networks can sometimes be associated with a more stable community structure, where groups of interacting species form functional guilds [36]. Further, the low centralised network suggests the skin microbial community is resilient to external environment changes, including transient microbes. It is also worth noting that some of the most abundant core bacteria were not the most connected members of their respective networks. This suggests that high abundance does not necessarily equate to a central structural role in the community.

However, the positive relationship between the gut and skin microbial compositions of *I. bannanicus* larva highlights how gut microbiota could affect the structure and compositions of the skin microbial community through immune pathways [37]. Furthermore, many bacteria between the gut and skin showed significant correlations, suggesting their complex interactions affect the host’s health status.

The most interesting distinction, however, arises when comparing the caecilian larvae to anurans. The dominance of genus *Laribacter* consisted of *L. hongkongensis* in the caecilian gut, not in anurans. This bacterium is associated with community-acquired gastroenteritis [38]; this suggests the *I. bannanicus* larva might exhibit relatively weaker resistance to this bacterial pathogen. One possible reason is that our captivity affected their resistance via the changed gut microbial community. The effect of captivity on developing biota is a very important caveat. However, most core bacteria from caecilian skin were shared with anurans compared to the gut region. This result could be attributed to their different diets affecting gut microbiota, in which the carnivorous diet of *I. bannanicus* larvae is rich in protein and fats. In contrast, most anuran tadpoles have a predominantly herbivorous diet [39].

It is important to acknowledge the limitations of this study. The *I. bannanicus* larvae were maintained in captivity before sample collection, so captive conditions and a chironomid larvae base diet in captivity may have influenced their microbiome somewhat. In contrast, the anuran tadpoles were wild-caught from various habitats. Furthermore, faecal samples are only a proxy for the gut microbiome and may not fully represent the communities attached to the gut lining [40]. Future research should aim to sample wild caecilian larvae directly from their natural habitats to provide a more accurate picture of their microbial ecology.

Finally, this work provides the first characterisation of the gut and skin microbiomes of a caecilian larva. We show that these communities are distinct from each other and are shaped by both the local body site and, most significantly. This carnivorous diet sets them apart from herbivorous anuran tadpoles. This research fills an important knowledge gap for an understudied amphibian order and provides a foundation for future investigations into the ecology, evolution, and conservation of these unique animals.

## 5. Conclusions

We characterised the microbiome in the gut–skin axis of *I. bannanicus* larvae, finding that the community composition, including core microbiota, was distinct between faeces and skin. The main core bacteria for faeces were the genus *Laribacter*, one unclassified genus from Bacteroidota, and *Akkermansia*, while the genus *Flavobacterium* was for skin. The bacterial community showed higher diversity and more complex relationships in skin than those on faeces. However, the microbial compositions show significant relationships between skin and faeces. *I. bannanicus* larvae share many bacteria with larval anurans but also have some specialised core bacteria.

## Figures and Tables

**Table 1:** Core bacteria in faeces and skin of *I. bannanicus*. Bold fonts indicate the core ASVs of *I. bannanicus* that presented in anurans.

Region	Feature ID	Taxon	Relative Abundance (%)
	ASV9	p__Proteobacteria; c__Gammaproteobacteria; o__Burkholderiales; f__Aquaspirillaceae; g__Laribacter	7.71
	**ASV10**	p__Bacteroidota; c__Bacteroidia; o__Bacteroidales; f__Unclassified; g__Unclassified	6.72
	**ASV12**	p__Verrucomicrobiota; c__Verrucomicrobiae; o__Verrucomicrobiales; f__Akkermansiaceae; g__Akkermansia	6.25
	ASV15	p__Bacteroidota; c__Bacteroidia; o__Bacteroidales; f__Unclassified; g__Unclassified	5.19
Faeces	ASV39	p__Firmicutes; c__Clostridia; o__Clostridiales; f__Clostridiaceae; g__Clostridium_sensu_stricto_1	1.75
	**ASV59**	p__Proteobacteria; c__Gammaproteobacteria; o__Burkholderiales; f__Sutterellaceae; g__Unclassified	1.07
	ASV73	p__Bacteroidota; c__Bacteroidia; o__Bacteroidales; f__Rikenellaceae; g__dgA-11_gut_group	0.92
	**ASV90**	p__Bacteroidota; c__Bacteroidia; o__Bacteroidales; f__Tannerellaceae; g__Parabacteroides	0.72
	ASV114	p__Bacteroidota; c__Bacteroidia; o__Bacteroidales; f__Unclassified; g__Unclassified	0.58
	**ASV115**	p__Proteobacteria; c__Gammaproteobacteria; o__Enterobacterales; f__Enterobacteriaceae; g__Escherichia-Shigella	0.18
	ASV254	p__Bacteroidota; c__Bacteroidia; o__Bacteroidales; f__Bacteroidaceae; g__Bacteroides	0.16
	ASV268	p__Firmicutes; c__Clostridia; o__Oscillospirales; f__Butyricicoccaceae; g__Unclassified	0.14
	ASV6	p__Bacteroidota; c__Bacteroidia; o__Flavobacteriales; f__Flavobacteriaceae; g__Flavobacterium	8.7
	**ASV16**	p__Proteobacteria; c__Gammaproteobacteria; o__Burkholderiales; f__Comamonadaceae; g__Unclassified	3.82
	**ASV17**	p__Proteobacteria; c__Gammaproteobacteria; o__Burkholderiales; f__Comamonadaceae; g__Ideonella	4.06
	**ASV29**	p__Proteobacteria; c__Gammaproteobacteria; o__Salinisphaerales;f__Solimonadaceae; g__Hydrocarboniphaga; g__Unclassified	2.06
	**ASV49**	p__Proteobacteria; c__Gammaproteobacteria; o__Burkholderiales; f__Comamonadaceae; g__Unclassified	1.11
	**ASV103**	p__Proteobacteria; c__Alphaproteobacteria; o__Rhizobiales; f__Rhizobiaceae; g__Unclassified	0.51
Skin	**ASV115**	p__Proteobacteria; c__Gammaproteobacteria; o__Enterobacterales; f__Enterobacteriaceae; g__Escherichia-Shigella	0.25
	**ASV124**	p__Bacteroidota; c__Bacteroidia; o__Cytophagales; f__Spirosomaceae; g__Emticicia	0.48
	**ASV129**	p__Proteobacteria; c__Gammaproteobacteria; o__Burkholderiales;f__Rhodocyclaceae; g__Methyloversatilis; g__Unclassified	0.49
	**ASV136**	p__Proteobacteria; c__Gammaproteobacteria; o__Pseudomonadales;f__Moraxellaceae; g__Perlucidibaca	0.48
	**ASV137**	p__Proteobacteria; c__Gammaproteobacteria; o__Salinisphaerales; f__Solimonadaceae; g__Nevskia	0.37
	**ASV173**	p__Proteobacteria; c__Gammaproteobacteria; o__Burkholderiales; f__Comamonadaceae; g__Paucibacter	0.21
	**ASV174**	p__Proteobacteria; c__Gammaproteobacteria; o__Alteromonadales; f__Alteromonadaceae; g__Rheinheimera	0.28
	**ASV176**	p__Proteobacteria; c__Gammaproteobacteria; o__Burkholderiales; f__Comamonadaceae; g__Acidovorax	0.17
	**ASV228**	p__Proteobacteria; c__Alphaproteobacteria; o__Caulobacterales; f__Caulobacteraceae; g__Brevundimonas	0.16
	ASV255	p__Proteobacteria; c__Gammaproteobacteria; o__Burkholderiales; f__Comamonadaceae; g__Hydrogenophaga	0.14
	**ASV293**	p__Proteobacteria; c__Alphaproteobacteria; o__Rhizobiales; f__Rhizobiaceae; g__Unclassified	0.12

**Table 2:** Topological features of bacterial community network across faecel and skin of *I. bannanicus*.

	Nodes	Edges	Average Degree	Average Path Length	Graph Diameter	Graph Density	Clustering Coefficient	Betweenness Centralization	Degree Centralization	Modularity
Faeces	79	303	7.6709	1.8680	5	0.0983	0.6218	0.0321	0.2863	0.2342
Skin	81	437	10.7901	3.2509	11	0.1349	0.8069	0.1145	0.2276	0.2521

## Data Availability

The raw metagenome data is being uploaded at the National Centre for Biotechnology (NCBI) with the BioProject accession numbers PRJNA764182 and PRJNA1280095.
